# Temporal development of high-performance translational teams

**DOI:** 10.1017/cts.2023.545

**Published:** 2023-05-15

**Authors:** Allan R. Brasier, Shannon L. Casey, Felice Resnik, Betsy Rolland, Elizabeth S. Burnside

**Affiliations:** 1 Institute for Clinical and Translational Research, School of Medicine and Public Health, University of Wisconsin-Madison, Madison, WI, USA; 2 Carbone Cancer Center, School of Medicine and Public Health, University of Wisconsin-Madison, Madison, WI, USA

**Keywords:** Translational teams, development model, design for dissemination, transactive memory system, shared mental model

## Abstract

Successful translation involves the coupled application of knowledge**-**generating research with product development to advance a device, drug, diagnostic, or evidence-based intervention for clinical adoption to improve human health. Critical to the success of the CTSA consortium, translation can be more effectively accomplished by training approaches that focus on improving team-emergent knowledge skills and attitudes (KSAs) linked to performance. We earlier identified 15 specific evidence-informed, team-emergent competencies that facilitate translational team (TT) performance. Here, we examine the SciTS literature describing developmental, temporal dynamics, and adaptive learning stages of interdisciplinary teams and integrate these with real-world observations on TT maturation pathways. We propose that TTs undergo ordered developmental phases, each representing a learning cycle that we call *Formation*, *Knowledge Generation*, and *Translation*. We identify major activities of each phase linked to development goals. Transition to subsequent phases is associated with a team learning cycle, resulting in adaptations that enabling progression towards clinical translation. We present known antecedents of stage-dependent competencies and rubrics for their assessment. Application of this model will ease assessment, facilitate goal identification and align relevant training interventions to improve performance of TTs in the CTSA context.

## Introduction

The success of the interdisciplinary team in terms of productivity, commercialization, and social impact has fueled a revolution in the approach to 21^st^ century science [1]. The Clinical and Translational Sciences Awards (CTSAs) seek to develop, test, and disseminate interventions to enhance translation to the clinic. This process has resulted in the emergence of *translational science* as a nascent discipline—a standardized knowledge base for enhancing the application of best practices to prevent disease or improve health [2]. One successful strategy used in this discipline has been by identifying, adapting, and applying relevant best practices from the broader science of team science (SciTS) [3–6] to define processes and practices supporting an effective Translational Team (TT). The TT approach provides a feasible solution for enhancing translation of effective health interventions into practice, providing a strategy for addressing the complexity, funding limitations, reproducibility, and regulatory challenges inherent in clinical research [7,8].

**Figure 1.**
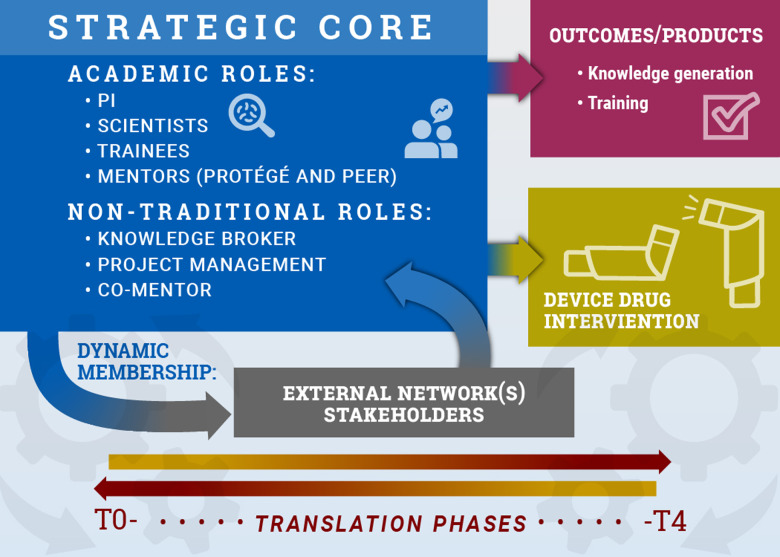
**The translational team (TT) model.** A schematic of the strategic core of a CTSA-type TT. The strategic core includes the personnel involved in the translational research across its lifespan, whose integration and effective interactions are essential for team success. These members include traditional academic roles [such as the principal investigator, early career trainee (e.g., a CTSA-funded KL2 scholar), research scientists] and those in nontraditional roles (knowledge brokers, project managers, and mentors). During the conduct of translational research, the strategic core interfaces with external scientific and professional networks, including scientific societies, professional societies, and clinical research programs. In addition, external stakeholders (patient advocacy groups, industry partners, community groups) also play important roles at various stages of translation. As the TT advances across the phases of the translational spectrum, from preclinical (T0) to clinical and community adoption (T4), the TT generates two major outcomes. Two types of outcomes are knowledge generation and training, characteristics of academic knowledge-generating teams. Another outcome is development of a drug/device/intervention, characteristic of an industry product development team. Reproduced from [14], with permission.

TTs are a hybrid of an academic knowledge-generating team and an industry-like product development team adapted to span the diverse domains of translational research from preclinical development to adoption into practice [3]. Specifically, a TT is composed of a diverse, dynamically engaged membership that interacts, adapts, and evolves to advance a product (device/drug/diagnostic) or evidence-based intervention (process or behavioral intervention) toward clinical or community implementation to improve human health (Fig. 1, [3–5]).

TTs are distinct from generic interdisciplinary teams in their construction, clinically focused taskwork, academic environment, and dynamic, voluntary membership. As the factors that influence performance of TTs become known, support for effective TTs can be optimized. To this goal, earlier work by a Team Science Affinity Group identified five interdependent, team-emergent competency “domains” influencing CTSA-type TTs performance [4]. These domains are (1) ***affect***, a domain describing that the bonds between TT members grounded in a concern, empathy and shared regard for others [9]; (2) ***communication***, a state where the TT effectively exchanges information and integrates team member expertise to solve research problems [10]; (3) ***management***, a term referring to leadership actions that effectively organize and sustain components of multicomponent investigation [11]; (4) ***collaborative problem solving***, a process where cognitive and social skills of the TT are used to integrate research findings and discipline-grounded interpretations into a cohesive model [12]; and (5) ***leadership***, the process of providing or supporting the cognitive, resource, and affective needs for a TT [13]. This work advanced the concept that team-emergent competencies arise from member interactions beyond the behaviors of individuals participating on the team.

Extending the work on TT competency domains, we identified 15 specific, team-emergent KSAs whose mastery of which are associated with high-performance TTs through a scoping literature review [14]. A mapping and description of specific competencies to the broader competency domains are shown in Table 1. This work focused on the primacy of a “triad” of KSAs: an inclusive environment, openness to transdisciplinary knowledge-sharing, and situational leadership. The literature indicates that the contemporaneous practice of the KSAs in this core triad serves to powerfully reinforce competency attainment in other domains. Despite the recognition that these competencies were associated with high-performance teams, how these competencies develop and evolve from team-level interactions are currently under-addressed questions. In this study, we use a scoping literature review to examine team development theories applicable to empiric observations of how TTs mature over time and align these with observational studies of the TTs within the CTSA environment.

**Table 1.** Translational team (TT) competency matrix

Domain	Competency	Description
Affect	**Building** **trust**	Creating team environment where members rely on others because they are accountable, responsible, and will support others.
	**Psychological safety**	The perception that taking risks in challenging interpretations, scientific dogma, or team processes is acceptable and has no consequences.
	**Cohesion**	The strength and extent of interpersonal connections between team members.
Communication	**Knowledge sharing**	Team members provide other members with technical information, “know-how” and skills relevant to advancing the team’s translational product.
	**Transactive memory system**	A group-level knowledge of “who” on the team has “what” expertise.
Management	**Team membership** **Roles and responsibilities**	Managing the research expertise needed to address current research questions of the TT.
	**Shared Visioning**	This shared, organized understanding and mental representation of knowledge of the team’s goal (including shared mental model).
	**Project management**	Coordination of team activities, learning goals.
Collaborative problem solving	**Adaptive behaviors**	The process by which teams respond to changes in their environments by modifying their processes and goals.
	**Collective intelligence**	Shared group-level knowledge and skills are used for consensus decision-making.
	**Transdisciplinarity**	Problem-solving activity drawing in perspectives—from diverse team members—that transcend traditional scientific boundaries.
Leadership	**Conflict resolution**	Management of task and person-based inter-team conflict.
	**Sensemaking**	The process of providing insight into an unexpected disruption to productive activity.
	**Networking**	Establishing interpersonal and scientific ties with external collaborators and networks.
	**Goal setting**	Process of setting clear and challenging goals that motivate members to acquire new skills or achieve performance targets.

Shown are the TT competency domains [4]; specific competencies supporting each [14] are also listed, with a description of knowledge, skills, and attitudes.

Development models describe the processes that generic interdisciplinary teams go through from their initial conception to eventual disbandment [15]. These models are valuable because they provide a conceptual framework for explaining how teams develop and they inform stage-relevant, team-focused training strategies for enhancing performance. Development models have been used for understanding knowledge-generating teams, product-development teams, and teams in extreme environments [16]. However, each of these team types has characteristics that are distinct from those of a TT. These unique features suggest that developmental models for optimal TT performance need to account for these important differentiating characteristics. For example, members of industrial new product-development teams are empaneled based on individual skill sets and principally focused on product development [3,17]. Members may not have extensive prior knowledge of the group members and their membership only persists throughout the lifecycle of the team until product delivery. By contrast, membership in a TT is within an academic environment, scientist participation is voluntary and the team activities are focused on both knowledge generation and product development [3,18]. Additionally, TT members often have prior knowledge of team members. In contrast to generic knowledge-generating teams that conduct short-term, time-bound planning, and decision-making focused on a single organizational problem [19], TTs can operate over a much longer time frame, conduct multiple projects at various stages simultaneously, and are responsible for implementing a product-like clinical intervention. Most importantly, successful TTs are learning and adapting over time [14], conducting cycles of introspection and goal realignment, often triggered by external factors, such as advances in the scientific or medical fields in which they operate.

**Figure 2.**
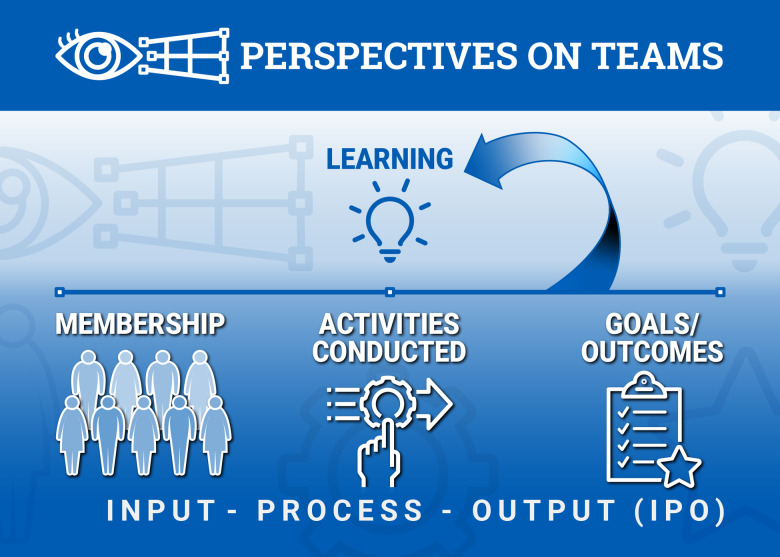
**Learning perspectives on teams.** Schematic view of input–process–output (IPO) conceptual model of team productivity. The IPO model suggests that many factors (inputs) influence a team’s activities (processes) that result in outcomes (knowledge generated and products developed). In this manuscript, we view team development from a team-based learning lens, that arises from these activities. Viewing translational teams (TTs) from a learning perspective has an important advantage over sequential IPO development models because learning models identify which team competencies are most important and when application of these skills will have the most impact. This model can be used in the assessment of team progress and provides guidance on stage-relevant, team-focused training for promoting TT performance.

In this work, we examine team development models and taxonomies of team development or maturation from the broader Science of Team Science (SciTS) field and adapt these to empiric studies of TTs in a CTSA environment [5]. Instead of approaching team maturation from an Input-Process-Output (IPO) view, we instead focus on team-based learning (Fig. 2). The focus on team learning is based on the axiom that TTs are continuously adapting and evolving in a complex and changing environment. For the purposes of this work, we define team learning as a change in the TT’s collective knowledge state [20]. Using this definition, team learning can be observed by convergence in the teams shared mental model (SMM), a term referring to the team’s collective understanding of information content or structure [21], or its Transactive Memory System (TMS), a term referring to shared information of “who” on the team knows “what” [22]. Team learning results in an adaptation, an outcome where the TT processes are better suited for achieving the goals of the team. Viewing TTs from this learning perspective has an important advantage over sequential IPO development models because learning models identify “which” team competencies are most important and “when” application of these skills will have the most impact. This model can be used in the assessment of team progress and provides guidance on stage-relevant, team-focused training for promoting TT performance.

## Methods

A scoping literature review was conducted in the Medline Core Collection to update literature identified in prior reviews from 2010 to 2022 according to the scoping review protocol (Supplementary File S1). From 78 citations (Supplementary File S2), 10 abstracts were selected for those that were (1) Empiric-observational; Empiric-survey, Meta-analysis, or Expert opinion/panel studies; (2) Included analysis or description of developmental phases; and (3) were relevant to Knowledge-generating, product development, innovation, or translational teams. We considered taxonomies of sequential team development and learning theory (outcomes and processes) to build these developmental phases. Vignettes are deidentified descriptions from TTs observed at the University of Wisconsin CTSA under IRB 2017-0860-CP007 (Renewed 6/27/2022).

## Results

### Models of Team Development

Sequential development models describe a series of stages where teams accomplish particular goals within each stage and transition to the next. A seminal example of a sequential developmental model that has informed the field of team effectiveness research for over 60 years is Tuckman’s model of forming, storming, norming, performing [23], and ultimately adjourning [19]. This model was formulated by analysis of 26 group development studies that were primarily focused on socioemotional structures and task activities. The Tuckman model focuses on two parallel team processes: the socio-emotional structure within the team and task behaviors exerted by the team. During the storming and norming phases, the social–emotional aspect plays a central role, whereas in the performing, when socio-emotional issues are largely addressed, the task activity is predominant.

Drawing from group dynamics and organizational behavioral research, a sequential four-phased linear developmental model for generic transdisciplinary research has been proposed more recently [24]. This model consists of the following phases: (1) Development, a phase where the group explores the problem space and identifies the disciplines that need to be involved; develops an approach, shares a mission and goals, and establishes trusting relationships. (2) Conceptualization, a phase where the group develops research questions, formulates hypotheses, establishes a conceptual framework, and plans a research design that integrates collaborators' disciplinary perspectives and knowledge domains. (3) Implementation, a phase where the research program is launched, conducted, refined, and the individual roles of team members are clarified as additional new members are engaged and integrated into the team. (4) Translation, a phase where the team applies research findings to advance progress along the discovery–development–delivery continuum to ultimately provide innovative solutions to real-world problems. In this model, outcomes are to provide a bridge to animal or human studies that have broader societal impact. The four phases of the disciplinary model are thought to be primarily sequential, where all phases must be encountered by the team and in a linear manner, although the model allows for recursive steps to occur.

### Findings From Observational Studies of TTs in CTSA Environment

**Figure 3.**
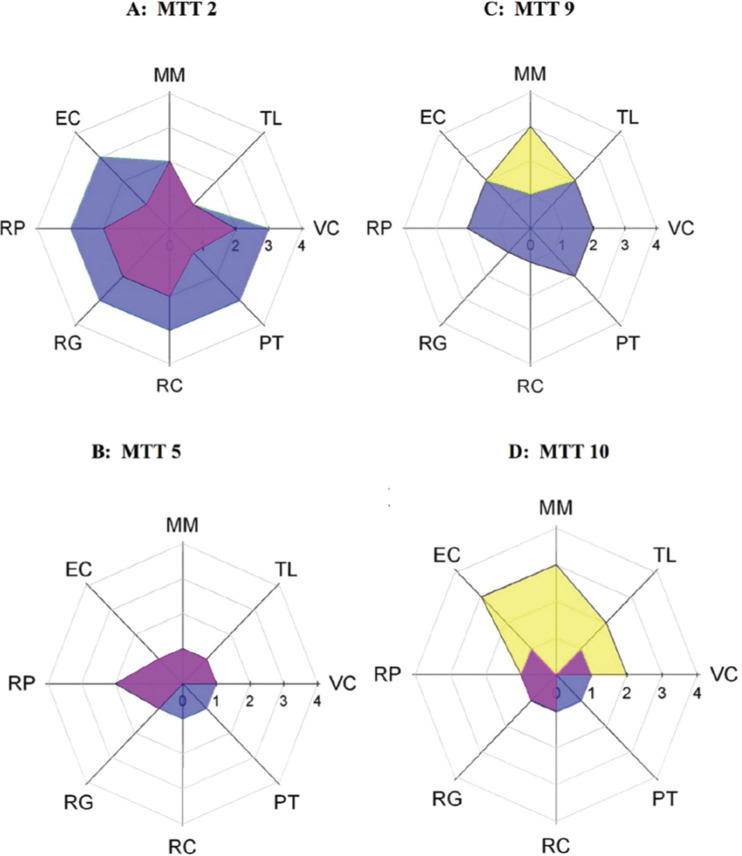
**Stochastic growth of translational teams (TTs).** Radar graphs depicting growth of four exemplar teams. Axes represent four research output factors and four team process factors assessed by independent observers in TTs in a CTSA environment. Outcomes are EC, external communication/collaboration; MM, meeting management; TL, transformative leadership; VC, vision and charter; PT, progress in translation; RC, research communication and program growth; RG, research generation; RP, research plan. Data from 2011 are shown in yellow and data from 2013 are shown in purple. Areas of overlap, which represent outcomes that have been maintained or improved in 2013 versus 2011, are shown in magenta. Note that TTs grow in different dimensions and to different extents. Reproduced with permission from [5].

**Figure 4.**
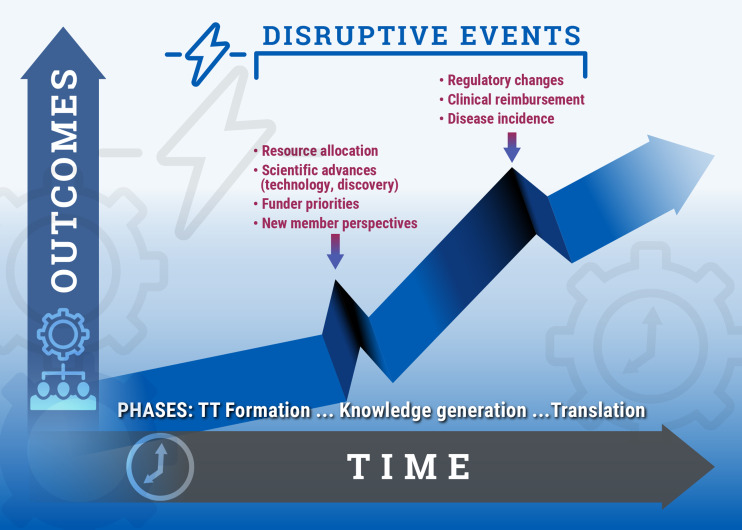
**Disruptive events in translational team (TT) maturation.** Inherent in the evolutionary learning model, team outcomes (publications, grants, intellectual property, interventions) do not accumulate in a linear process, but are marked by disruptive events serving as “critical moments” or “transition points.” These external events are shown by vertical arrows. Shown are two such external transition points associated with transition from *formation* to *knowledge generation,* and the transition from *knowledge generation* to *transition.* Transition points promote adaptive evolution of the TT, stimulating new collective knowledge and, in some cases, causing transition to the next phase of evolution.

Although the linear sequential model of transdisciplinary research is conceptually intuitive, we examined whether this model is applicable to TT development in the CTSA environment (see Methods and Supplementary File 1). We re-examined a mixed methods assessment of temporal features of 10 TTs composed of over 100 members in an academic CTSA environment [5,6]. A rubric to observe team evolution was developed, assessing team development in two major dimensions: (1) Capacity and (2) Productivity. The first domain, capacity, is a measure of effective team processes. These processes include the development of a shared vision, efficient meeting management, transformational leadership, and communication. The second dimension, productivity, is an outcome-based measure of how the team transitions across the translational spectrum. Productivity included the development of a mature research plan, production of research output (manuscripts, products), and societal impact of its translational intervention [5,6]. Relevant findings included that not all teams matured at the same rate across both dimensions or in the same way. A radial diagram from this study illustrates the point (Fig. 3). One team showed substantial growth in capacity (research generation, research production, external collaboration, and vision, Fig. 3a). In contrast, another team advanced only in development of its research plan (Fig. 3b). Importantly, for all of these TTs, productivity was not uniformly linear, but punctuated by sudden changes in new concepts, product development, and resources/funding. These punctuated, unpredictable/stochastic changes should be incorporated into any model for assessing and supporting TTs in an academic environment (generic disruptive changes are schematically illustrated in Fig. 4).

### Limitations of Sequential Linear Models

Although the four-phase sequential model of transdisciplinary research provides a useful framework for enhancing efficiency in generic transdisciplinary research teams, the empiric finding that team maturation is stochastic makes problematic the application of sequential models that assume development is the result of a predictable, fixed order of phases [15]. This limitation obscures the ability to measure the effectiveness of interventions on improving the performance of TTs in the CTSA environment.

Another important limitation of the four-phase sequential model is that the phases of team development are not clearly separable in real-world TTs. For example, hypothesis generation, a characteristic that distinguishes conceptualization from the development phase, in reality, occurs very early in TT development. In our observations of TTs in academic environment, TTs develop their membership and research hypothesis concurrently, not sequentially. Additionally, CTSA-type TTs conduct multiple research projects simultaneously, which are at different stages of maturation, making the application of a single sequential model difficult (See Vignette, Box 1).

Box 1. Formation – Knowledge Generation Transition Vignette

**Translational Product/Intervention:** Advance therapeutics and device for treatment of fibrosing interstitial lung diseases (ILD).

**Background:** Interstitial lung disease (ILD) refers to a collection of diseases without known cause that produce progressive scarring (fibrosis) of the lung. Over 250,000 patients carry the diagnosis with 50,000 new cases diagnosed annually. No treatments are available that reverse the course of disease. Due progressive nature of ILDs, the overall healthcare burden is high with decreased healthcare quality of life, and increased healthcare utilization, resulting in up to 40,000 deaths annually. Additionally, the incidence of ILD is rising sharply in the US in general and due to ILD produced by severe COVID-19 respiratory disease.

**Approach:** An investigator in the Department of Medicine (P1), expert in molecular and cellular biology, sought to advance a small molecule therapeutic targeting a pathogenic pathway driving lung remodeling and repair. A collaborator expert in nanoparticle formulation from the School of Pharmacy (P2) and a collaborator expert in noninvasive imaging of fibrosis from the School of Engineering (P3) were approached.

**Activities**

- **Developing roles:** A small animal model was selected by P1. Formulation was developed through iteration of lab of P1 and P2. P3 adapted light interference microscopy
- **Integrating disciplines:** A series of meetings were held where each discipline presented their approaches to the problem.
- **Hypothesis generation:** the group developed a shared hypothesis that epigenetic regulator of epithelial cell stress drove myofibroblast activation.
- **Trust building:** The team participated in Collaboration Planning, developing a authorship agreement, increasing the trust of the team member in participating in shared research.

**Transition to Knowledge Generation:** based on feedback from research proposals from Foundation grants and institutional pilots, resources were obtained to enable the transition of the project to *Knowledge Generation*.

**Projects:** Three projects were initiated towards the development and application of nanoparticle therapy for treatment of fibrosis: 1) Medicinal chemistry optimization of nanoparticle for encapsulation targeting regions of fibrosis (P1+P2); 2) Miniaturization of probes for light interference microscopy imaging in human airways (P3+P2); 3) Standardization of animal models of IPF for preclinical efficacy testing and validation of imaging technology (P1 + P2 +P3).

Additionally, the four-phase sequential model is insufficiently developed for inclusion of Dissemination Science for high-performance TTs in the CTSA environment. Although the model applies the term “translation” to application of research generically for societal benefit, the explicit inclusion of dissemination and implementation (D&I) science is omitted. D&I is the process of applying the most effective strategies to successfully disseminate, implement, and sustain evidence-based/effective practices in real-world settings [25,26]. To be effective, D&I is a systematic approach that teams must take for effective utilization of translational interventions. This approach includes (1) stakeholder involvement early in the process of translational intervention design (stakeholders include patients, funders, advocacy groups, and purveyor organizations); and (2) application of the principles of Design for Dissemination and Sustainability (DDS) to enhance the fit between a health program, policy, or practice and the context in which a translation is intended to be adopted. Without this fit, beneficial translations are unsustainable in the health care environment and abandoned. We contend that D&I is sufficiently distinct in its approach to merit explicit designation in relevant developmental models of high-performance TTs in the CTSA environment.

Most importantly, the four-phase sequential model focuses on stage-relevant activities but does not describe how team-emergent competencies arise. These specific competencies have a robust evidence base associated with high-performance TTs in the CTSA environment ([5] and reviewed in [14]). Without understanding when team-emergent competencies are needed or arise, team training-focused interventions may not be directed when they could have maximal impact.

### Viewing Team Development Through an Evolutionary Learning Perspective

Team development refers to progressing the state of maturation of the team knowledge and product across the translational spectrum (Fig. 4). Knowledge generation and translational advances are nonlinear; their course is punctuated by unanticipated research findings, outside discoveries, and/or changes in extramural funding priorities. These disruptive events serve as a “critical moment” or “transition point” in punctuated equilibrium theory [27] that triggers a team learning episode [28] (Fig. 4). Defined earlier, a team learning episode refers to a shift in the teams SMM or its TMS, resulting in a process improvement within the team. Effective TTs are continuously adapting to new challenges through this process. Studies of the core processes and emergent states underlying team learning [29–32] have found a strong positive, causal, relationship between team learning/adaptation and team performance [33]. This finding suggests that focusing on promoting team learning behavior will impact TTs. Of specific relevance to the translational product developed by TTs, team learning has a strong positive effect on the innovativeness and speed to market of the new products [34].

Team learning episodes are discernable periods where teams become aware of problems or are subjected to disruptive events. Team learning processes involve team members seeking new information, gathering feedback, and collectively interpreting this information for use in subsequent performance cycles [28]. Through this activity, TT members evaluate the outcome, examine past performance, and develop plans for next steps, resulting in collective learning and adaptation [28]. Importantly, each step in the phases of team development can be viewed as a learning or performance episode [35].

In contrast to the linear development models discussed above, an “unfolding” evolutionary learning model has been proposed [20]. The unfolding evolutionary learning model emanated from a comprehensive literature survey that proposed *how* teams learn, *what* they learn, and *when* they do it. This model extended the well-known Gersick “punctuated equilibrium model” [27] in two important ways: (1) Evolutionary learning accounts for the fact that teams are engaged in multiple projects simultaneously, often at different stages of maturity; and (2) Evolutionary learning incorporates our empiric findings that TTs are subjected to multiple transition points throughout their maturation arising from external disruptors leading to learning cycles (schematically shown in Fig. 4), rather than a single transition point arising after a period of inertia, as proposed by Gersick.

This unfolding evolutionary model is an appropriate development model of TTs because this model: (1) incorporates phases of team development consistent with empiric observations of TTs [5]; (2) accounts for multiple, stochastic transition points that promote team learning and adaptation (see *Vignettes in* Boxes 1 and 2); (3) embraces the empiric findings that TTs are engaged in multiple projects simultaneously (see Box 1; and [3]); and (4) enables understanding of what knowledge skills and attitudes (KSAs) are needed at each phase of team development associated with high-performance TTs. Consequently, we maintain that the unfolding evolutionary learning model provides a relevant foundation for formulating a TT development model. For simplicity, we will subsequently refer to this model as an “evolutionary learning model.”

Box 2. Knowledge Generation -Translation Vignette

**Translational Product/Intervention:** Revise an evidence-based falls prevention program to enhance reach and efficacy for Hispanic/Latino older adults, packaging it for broad dissemination

**Background:** Falls in the elderly are an important cause of morbidity, loss of independence, resulting in injuries and mortalities. One in four adults over the age of 65 fall annually; resulting in 3 million older people treated in emergency departments for fall-related injuries. While the risk of falling is similar across race and ethnicity, the age adjusted death rate due to falls has been climbing for Hispanic/Latino seniors. Additionally, there are limited evidence-based fall prevention programs designed for Hispanic/Latino seniors.

**Approach:** A team that worked together on an evidence-based training intervention shown to reduce the risk of falls, realized the need to adapt the program to develop a culturally and linguistically tailored program for Hispanic/Latino seniors.

**Knowledge Generation Activities**

- New member onboarding:
  - New team members invited to bring valuable perspectives to enhance cultural adaptation.
  - Team now consisted of the PI, implementation scientists, senior center community members and leaders, program facilitators, WI Institute for Healthy Aging (WIHA), Community Academic Aging Research Network (CAARN), WI Aging Network (WAN),.
  - To onboard new members, a series of meetings were held to integrate new members into the team, provide them with a mental model of what has been done, where the team wants to go, and what the team aims to achieve.
- **Knowledge sharing:** Additional meetings were held after major milestones to de-brief and share experiences.
  - Team members shared difficulties and successes. By sharing learning, the team better understood systems, identifying key elements and adaptations necessary for success of this adapted program.
  - Investigators shared stories of previous actions and learning to emphasize their humility, openness to new ideas, and understanding the importance of an iterative process to adapt the program, which required open discussion and exchange of knowledge.

**Transition to Translation Phase:** Based on feedback from stakeholders and results from piloting Pisando Fuerte, the project was able to transition to *Translation*.

**Translational Activities:**

- **Adaptation:** Evaluation of the pilot implementation of PF highlighted aspects that threatened dissemination: 1) overall program language level was too high for participants with limited literacy, and 2) facilitator training and background information for facilitators only in English was a barrier for facilitators and organizations. Both of which affected program fidelity. Facilitator training was translated to Spanish and tailored to include exercises and activities meeting sociocultural needs of bilingual facilitators and monolingual seniors.
- **Stakeholder engagement:** To enhance cultural tailoring of PF, additional stakeholders including senior centers (adopter organization), program facilitators (providers), CAARN (Academic-Advisory Network), WAN (Community group), and WIHA (Purveyor) provided feedback on trainings and program adaptation to meet the needs of all team members.
- **Design for Dissemination:** Involvement of the purveyor organization, WIHA, enabled refining PF. The updated program has been adopted by three community sites with several other sites sharing their interest in adopting the program.

**Translational Outcomes:**

The final version was submitted to the National Council of Aging (NCOA) for inclusion in their list of programs eligible for title IIID Older Americans Act funding.

### A Model of TT Development Based on Team Learning

**Figure 5.**
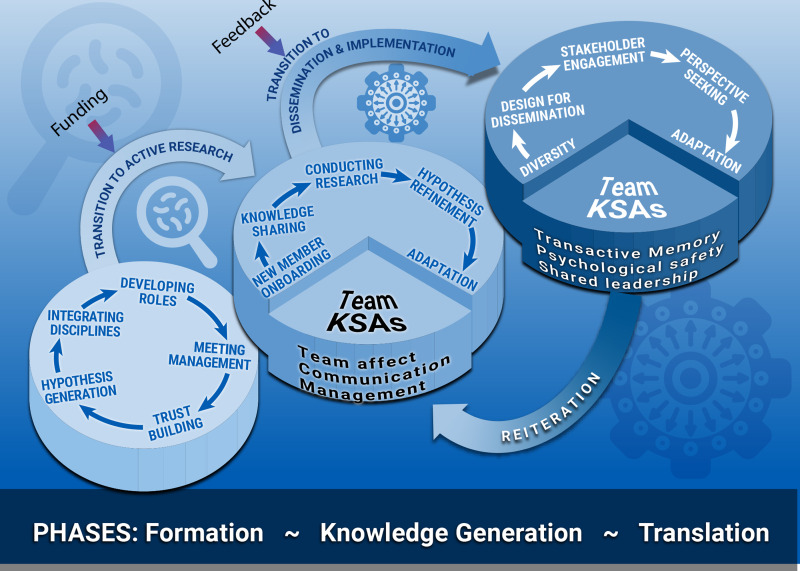
**Evolutionary learning model for translational team (TT) maturation**. Shown is a schematic model of the phases of TT development with external transition points. The team *formation* phase conducts activities focusing on enhancing team membership, establishing the basis of inter-team trust and developing a shared mental model. These are fostered by transformational leadership behaviors. As the team develops its hypothesis and acquires resources, it transitions into a *knowledge generation* phase. KSAs developed by the team in its *formation* phase support and are refined by activities in the *knowledge generation* phase. During *knowledge generation,* the team conducts activities in hypothesis testing, evaluation, and refinement. As the evidence base of effectiveness of the translation is established, the team transitions into the *translation* phase. KSAs (e.g., transactive memory systems, psychological safety and shared leadership) learned during the *knowledge generation* phase support activities in *translation,* including engaging new stakeholders, and purveyors. During translation, complex “Collaborative problem solving” leads to transdisciplinarity.

Based on the relevancy of the “unfolding” learning and limitations of sequential developmental models, we propose an adapted three-phase evolutionary learning model for TT maturation. Each phase conducts activities focused on the major TT goal from team formation (*Formation*), to conducting research (*Knowledge Generation*) to implementation in health care/community (*Translation*). Transitions to the next phase are dependent on collective team learning, sometimes initiated by “disruptive events” (Fig. 4). The collective learning response to these disruptive events gives rise to team-emergent competencies used by the team throughout its subsequent maturation; these KSAs are linked to performance as reviewed earlier [14] (Fig. 5). We will first describe the primary activities within each phase, followed by the team learning and competencies that arise from them.

In the first phase, “*Formation*,” initially leadership assembles and leads a group in a series of activities leading to a team approach to address a translational problem. Initially, leadership is responsible for engaging and integrating scientists from different disciplines, developing members roles, applying meeting management processes, and role modeling to build a trusting environment (Fig. 5). In this process, group co-creation arises. Through group interactions (meetings and retreats), group members form communication networks and develop a shared understanding of the team’s purpose and goals, co-creation of norms of interactions, and hypothesis development (Fig. 5).

The activities conducted by teams in *Formation* are fundamental to developing the competency domains of “Affect,” a team-emergent KSA of empathy, consideration, and rapport for other members [9], forming a “Communication” system, a KSA that integrates knowledge and information into collective team work and task work, and advancing practice of “Management” referring to organizing, planning, and executing components of a TT research program. Illustrated in Table 1, with specific competencies, these KSAs are specially relevant and associated with high-performance TTs [14].

Transition from the *Formation* phase is provided by an external trigger of funding or resource acquisition, leading to collective group learning (see *Vignette* in Box 1; Figs 4 and 5). Upon acquisition of resources, the team reflects on its hypotheses and goals, refines these to align with available resources and realigns with its experimental plan, completing a learning cycle. The TT then transitions to the next phase building—and applying in more sophisticated ways—the “Affect,” “Communication,” and “Management” competency domains. These KSAs are incorporated into the shared behavior of the team, supporting activities in the next phase.

In the second phase, “*Knowledge Generation*” (Fig. 5), team members engage in conducting research projects, sometimes multiple, involving interdependent task work. In the process of coordinating complex taskwork, team members develop an understanding of how research will be completed in the TT and, through interpersonal interactions, share individual knowledge with their teammates. During the process of conducting research, research findings result in hypothesis refinement and adaptation. New members are brought onboard.

Activities within the *Knowledge Generation* phase are supported by the team-emergent KSA domains developed earlier (Table 1). For example, inter-team “Communication” skills enable team members to have a deepened understanding of “who” on the team has “what” expertise, enabling the formation of a transactive memory system (TMS), itself linked to accomplishment [36] and ultimately high-performance teams [14]. As data and its interpretation are generated by the task work conducted in the *Knowledge Generation* phase, team members challenge, test, and explore assumptions. These activities are supported by advancing the specific “Affect” competencies of trust and cohesion (Table 1, Fig. 5).

Multiple team learning cycles may arise in the *Knowledge Generation* phase, depending on the complexity of the translational project being conducted. External triggers for new rounds of team learning are driven by unexpected experimental results, outside findings, or changes in funding (see *Vignette* in Box 2; Figs. 4, 5). These triggers produce introspection, goal realignment, or refinement of the hypothesis or translational product. These disruptive events are addressed by leadership behaviors in “sense-making,” where leadership provides a mental image of where the team is and where they are going to create an action plan in the face of uncertainty [37]. Consequently, KSAs developed from learning cycles in the *Knowledge Generation* phase include specific competencies of psychological safety, knowledge sharing, sense-making, and developing a TMS [14]. These team-emergent competencies are used, adapted, and further refined during the *Translation* phase.

In the third phase, “*Translation*,” TTs incorporate new stakeholder members, establish networks with health systems, providers and purveyor organizations, and respond to their input, expanding cognitive diversity. Application of DDS principles refines the translational product promoting adaptations and perspective-seeking may trigger additional transition points of learning (see *Vignette* in Box 2).

Team-emergent KSAs arising within *Translation* include “Collaborative Problem Solving,” a team-emergent KSA characteristic of high-performance TTs within psychologically safe environments and enhanced by cognitive diversity [14]. “Collaborative Problem Solving” involves integration of diverse intellectual practices, methods and biases into a common interpretation of observed phenomena [38], resulting in transdisciplinarity [39]. Substantial changes in perspective or the translational project may trigger re-iteration of the *Knowledge Generation* phase, illustrating the nonlinearity of TT development [5].

### Origins and Development of Team-emergent KSAs During TT Maturation

A recent scoping review of the SciTS evidence base identified 15 team-emergent competencies whose expert application leads to enhanced team performance (listed in Table 1) [14]. In this analysis, observable behaviors of the KSA were described along a continuum from “novice” to “expert.” It is axiomatic that each of these competencies develops as a result of team interactions and undergoes maturation and refinement before an “expert” level of proficiency is reached. In the following section, we consider antecedents of the competency and interdependence with competencies in other domains. Finally, we place these within the phases of the evolutionary learning model leading to a model for how the competencies mature as the TT develops.

*Affect* refers to the development of empathy, affiliation, and rapport between members on the basis of shared regard for the other members of the TT [4,9]. Within this domain, three specific complementary competencies linked to high performance are “trust,” the confidence that team members have in the abilities of their colleagues to do reproducible work, share results, and discuss their interpretations, “cohesion” the strength and extent of interpersonal connection between team members, and “psychological safety,*”* a shared belief that the team environment is safe for risk-taking, formulating opposing ideas, or challenging team assumptions [28].

Current evidence supports that maturation of these three specific components of “Affect” is distinct and depends on different member interactions and contexts. Specifically, during *Formation*, the team leader works with potential team members to establish norms for interactions, communication, and data sharing that are safe for exchange, hypothesis development, and discussing alternative interpretations. In this phase, trust is initially displayed as trust in leadership or select team members (Table 2). However, it is intra-team trust, not just that of trust in selected members, that is most highly linked to team performance [40]. Intra-team trust develops after trusting norms have been established by leadership behaviors. In addition, studies have shown that cohesion and satisfaction are antecedents to intra-team trust [41]. We therefore propose that intra-team trust evolves within interactions and task-work conducted during *Knowledge Generation* and *Translation* (Table 2).

**Table 2.** Maturation of competencies across translational team

		Phase of team evolution		
Domain	Competency	***Formation***	***Knowledge Generation***	***Translation***
Affect	Building trust	Members have trust in leadership	Members develop trust in each other	Psychological safety- members are comfortable challenging each other and scientific results
Comm	Knowledge sharing	Members share their relevant knowledge and its application to the project	Members share expertise with immediate collaborators	Knowledge sharing expands to collective team
	Transactive memory systems	Leader knows disciplines of group	Members know some of the expertise in the team	Members know major skills of all team members
	Shared visioning	Team members co-create a vision for the project	Members understand major goals	Members have coherent, shared vision
Management	Managing roles	Leader works to identify major roles	New roles emerge as interdependent research is performed and new members brought on board	Members expand their own roles, through challenging goals, enhancing capacity
	Diversity	Multiple scientific disciplines in group	Additional science and community members brought into team	Community, patient advocacy groups valued and have input
	Meeting management	Team fosters effective meeting management	Meetings are agenda-driven	Data sharing and analysis promotes reproducible research
Collab Problem Solving	Learning/adaptive behavior		Group react and respond to disruptive influences	Group learning from disruptive behaviors change processes
Leadership	Sense making		Leader promotes sensemaking in disruptive events	Team members participate in sense-making
	Goal setting	Members assigned immediate goals	Members challenged with developing new capacity	

For each team-emergent competency domain, specific competencies are tabulated with examples of their proficiency for the three phases of evolutionary team learning. Abbreviations: Comm, communication; Mgmt, management; Collab, collaboration.

Like the maturation of intra-team trust, development of psychological safety is a group-level construct [28], dependent on multiple team and interpersonal antecedents. A meta-analytic review of ∼ 5,000 groups examining the origins of psychological safety and their effectiveness established that leadership interactions and work environment were more important than individual personality characteristics, such as emotional stability and openness to experience [42]. Others have proposed that psychological safety arises from trusting interpersonal relationships developed with other team members, organizational norms, as well as with the team leadership [43,44]. Psychological safety is, therefore, based on group-level interpersonal relationships, particularly with the leader, a supportive work environment, and organizational norms. We argue that the appearance of this complex and robust KSA would emerge in actively interacting teams, after team *Formation* (Table 2).

*Communication* is a team-emergent competency domain that refers to the ability to integrate knowledge and expertise in team member interactions and in task work. Specific competencies within “Communication” include “knowledge sharing,” a behavior where team members provide other members with technical information, know-how and skills relevant to advancing the team’s translational product, and transactive memory system (TMS), a group-level understanding of knowledge of “who” on the team has “what” expertise. In a manner similar to the maturation of specific competencies within team “Affect,” competencies within “Communication” arise from interdependent taskwork activities conducted during the *Knowledge Generation* phase of team development. Information sharing has been most intensively studied in the organizational literature as this behavior provides competitive advantage for knowledge-based or technological organizations. This work has found that trust, an organization’s learning orientation, and positive interactions/cohesion with other team members, including reciprocity, are all enabling factors for effective knowledge sharing. Knowledge sharing is highly influenced by team leadership behaviors, particularly transformational leadership [45,46]. Knowledge sharing is foundational for more advanced forms of information sharing, knowledge management systems, where a team’s collective knowledge is shared within the larger organization or field [47]. Hence, maturation of knowledge sharing advances as the team’s cohesion and trust develop from information sharing, advancing to knowledge sharing, and then to knowledge management systems (Table 2).

A TMS refers to group-level knowledge, where team members know “who” on the team has “what” expertise [20], developed through reciprocal exchange and joint effort between individual team members on collaborative activities [36]. Establishing a TMS involves both information processing as well as group identification. Using a social cognition framework, Liao proposed that the quality of information sharing by multidisciplinary team members with their own professional view/identification plus an established sense of group identity are both antecedents for a TMS [48]. Hence, team “Affect” enables a TMS. A TMS is important as work shows that sharing group-level knowledge saves time in taskwork, particularly in dynamic environments, leading to goal accomplishment [36] and high performance [14].

***Management*** refers to the activities conducted in the organization, planning, and executing components of a TT project. Specific competencies include establishing a cognitively diverse membership and shared visioning promoted by project management practices. TTs in an academic setting are composed of a dynamic membership that engages with a strategic nucleus of the PI and core scientific expertise [3,14]. Engaging this membership and defining their roles and responsibilities are key activities starting in the *Formation* phase and continuing throughout the *Knowledge Generation* and *Translational* phases of the evolutionary learning model (Fig. 4). Specific to the evolutionary learning model, TTs in the *Translation* phase incorporate the principles and approaches of D&I Science. As a result, new stakeholders, caregivers, patients/patient advocacy groups, and community members become team members. The processes of how these members are engaged and brought on board mature with knowledge sharing and the convergence of a shared, organized understanding of the team’s SMM [49]. SMMs work cooperatively with TMS and are important for team effectiveness, especially when teams are faced with complex, dynamic problems engaging in complex interdependent tasks [50].

Although the group develops shared goals and understanding of the hypothesis during team *Formation*, a fully developed SMM will emerge later as the team conducts inter-dependent task work, as the TT undergoes learning cycles and refines its goals [49]. The interdependent research activity, research, hypothesis testing, and refinement activities conducted during the *Knowledge Generation* phase produce convergence of the SMM (Table 2).

***Collaborative problem-solving*** is a state where the cognitive and social skills of the team are combined to interpret research findings, resulting in a cohesive mental representation of the problem space, resulting in shared interpretations [38]. During the *Translation* phase, new stakeholders, caregivers, patients, patient advocacy groups, and community members can provide differences in interpretation, perspective, or information processing styles within a team known as cognitive diversity [51–53]. Cognitive diversity influences an emergent collaborative problem-solving property coined “collective intelligence” [54], highly predictive of a team’s ability to perform on a variety of knowledge tasks[54]. Collective intelligence transcends traditional scientific boundaries to jointly define a problem, conduct problem-solving activity, and draw in perspectives from diverse team members resulting in the emergence of a “transdisciplinary” research program [39,55]. Similarly, transdisciplinarity arises as translational boundaries are broken down with high-level interactions amongst the team members.

***Leaderships*** provide the cognitive, motivational, affective, and management processes to help the team thrive in a complex and dynamic environment [13]. Team leaders provide essential support throughout the lifecycle of a TT, including establishing membership, defining roles, setting expectations, resolving conflict, and goal setting. Substantial scholarship has shown how sources of leadership and impactful activities undergo adaptation [56]. During *Formation*, leadership is primarily contributed by a PI inspiring group vision, seeking diverse perspectives, and modeling inclusive behaviors emblematic of transformational leadership skills [57]. Underscoring its importance, our empiric studies have shown that high-performance CTSA-type TTs are distinguished by transformational leadership skills, sometimes shared between the PI and the early career trainee [5]. Other important leadership activities include providing feedback to members and promoting an environment of psychological safety. TTs with leaders that provide “goal-setting” outperform teams without such leaders [58,59]. “Sense-making” is another leadership activity that enables a team to productively respond to disruptive events [60–62], turning episodic disruption into productive activity by providing insight into the event and developing a path forward. “Sense-making” frames a mental image of where the team is and where they are going in order to create an action plan in the face of uncertainty, enabling productive responses to transition points. Leaders are the most important sense-makers who shape followers’ perceived meaningfulness of work-related issues [63], including goal commitment and team identification [45]. Feedback enables adaptation and enhances long-term performance [58,64,65].

## Discussion

An unintended consequence of the application of the sequential development models on team maturation has been the view that team processes are largely immutable in nature, and focused on outcomes, rather than learning [66]. However, our observational studies show that TTs are in highly complex environments. Successful TTs learn and adapt in response to multiple influences typically occurring in unpredictable sequence rather than in a logical linear fashion. Consequently, team processes, affect, and behavioral patterns emerge over time [67]. To our knowledge, this is the first analysis of temporal changes in TTs with a focus on team learning and team skills. Our approach is not to establish “the” model for temporal development of TT, but rather to identify a practical model that guides impactful team-level interventions for team-focused training at development stages when the intervention is most needed. Based on learning outcomes, we believe this model will aid just-in-time team-focused CTSA training and evaluation interventions.

In this work, we provide an alternative view of linear, sequential models using a novel evolutionary learning model of TT development. We contend this evolutionary learning-based model is relevant to real-world TTs because it embraces “critical moments” or transition points [27], accounts for multiple projects being conducted by TTs that have been observed empirically, and informs understanding of phase-relevant competencies associated with high-performance TTs. An important challenge to testing this model will be to understand how TTs learn. Team learning occurs in a stochastic manner that provokes team member introspection, gathering information and feedback, collective re-interpretation, and incorporation into new SMMs, refinement of a TMS, and/or process improvement [28]. More work will be required to understand adaptive learning processes in the team environment.

An important test of the evolutionary learning model will be measurement of SMM convergence or TMS development as a proxy to team learning. Some conceptual models have been developed for individual professional learning, such as the “Master Adaptive Learner” that fosters the development and use of adaptive expertise in the medical profession [68]. However, measurement of team learning will be challenging, especially because the stochastic nature of their events and repetitive measures may be required. For example, convergence of SMMs can be assessed using concept maps and repeated measurements; however, these repetitive evaluations may be burdensome to active TTs. Successful measurement of team dynamics in the real world requires an approach that integrates individual, team, organization, and environmental contexts [69]. Proactive and purposeful planning of operationalization of constructs allows for analysis of specific team characteristics and dynamics that most benefit advanced understanding of the effectiveness of team interventions. A priori discrimination of the purpose of the measurement data within context is critical—here our goal is to understand facilitators of team transformation and effectiveness over time.

We contend that this three-phase evolutionary learning model provides clarity of phase-relevant goals, relevant-real-world defined transition points, and origins of team-emergent competencies that are associated with successful TTs. This model assumes competencies that are most impactful at transitions. We recognize that more work will be required to fully understand the relative importance of team-based competencies. In addition, it is likely that not all teams will manifest the same levels of competencies at major transitions. Competency attainment is likely to be influenced by the skill of team leadership and the context in which the TT is working. The influence of academic culture and leadership behaviors are likely to be major factors. The influence of academic culture on TT success and motivation of members is an area that requires further study.

Our model allows multiple cycles of *Knowledge Generation*, with team’s collective understanding advancing and improving with each cycle. Real-world experience demonstrated that TTs may cycle back from *Translation* phase to conduct additional cycles of *Knowledge Generation*, incorporating lessons learned through implementation of the translational product in the community, hospital, or clinic setting (Box 2, and Fig. 5). Team-based learning may, therefore, become more advanced with each *Knowledge Generation* cycle. This learning may also explain why teams with previous experience working together are more effective in future work than nascent groups. In addition, onboarding of new members will introduce the opportunity to develop further the “Affect” and “Communication” domains.

An important implication of this work is that phase assessment of TT can be used to guide team-based interventions, including the source and type of leadership needed at each phase. Our earlier work defining KSAs of high-performing TTs provided evidence that leadership behaviors influenced the adoption and application of virtually all team-emergent competencies. More work will be required to understand the important leader behaviors at each phase of TT development and the optimal source of that leadership.

In summary, development models are valuable guides for assessing and designing training interventions at the team level. In this manuscript, we challenge the existing sequential linear model of transdisciplinary team development and propose a flexible, evolutionary learning model consistent with field observations of TT evolution. This work will guide team-level training interventions to a phase when this intervention will be most impactful.

## Supplementary material

For supplementary material accompanying this paper visit https://doi.org/10.1017/cts.2023.545. click here to view supplementary material
