# Supplementary material for: Temporal development of high-performance translational teams
Source: J Clin Transl Sci. 2023 May 15;7(1):e117. doi: 10.1017/cts.2023.545 (PMC10225261; doi:10.1017/cts.2023.545)
Supplement: Supplementary file 1 [file ctssup.zip › S2059866123005459sup001.pdf]

Brasier AR et al.
Supplementary File 2
Scoping Literature Search Results

1. Rolland B, Resnik F, Hohl SD, Johnson LJ, Saha-Muldowney M, Mahoney J. Applying the lessons of implementation science to maximize feasibility and usability in team science intervention development. Article. J Clin Transl Sci. 2021;5(1)197. doi:10.1017/cts.2021.826
2. Luke DA, Carothers BJ, Dhand A, et al. Breaking down silos: mapping growth of cross-disciplinary collaboration in a translational science initiative. Clin Transl Sci. Apr 2015;8(2):143-9. doi:10.1111/cts.12248
3. Amaro CM, Alderfer MA, Gerhardt CA, Wawrzynski SE, Goldish M, Long KA. Bringing Together a Transdisciplinary Team to Create and Advance a Shared Vision for Research and Support for Siblings of Youth With Cancer. J Pediatr Hematol Oncol Nurs. Jan-Feb 2023;40(1):34-42. doi:10.1177/27527530221121727
4. Read EK, O'Rourke M, Hong GS, et al. Building the team for team science. Article. Ecosphere. 2016;7(3)e01291. doi:10.1002/ecs2.1291
5. Halvorsen KE, Knowlton JL, Mayer AS, et al. A case study of strategies for fostering international, interdisciplinary research. Article. J Environ Stud Sci. 2016;6(2):313-323. doi:10.1007/s13412-015-0336-7
6. Sancheznieto F, Sorkness CA, Attia J, et al. Clinical and translational science award T32/TL1 training programs: Program goals and mentorship practices. Article. J Clin Transl Sci. 2022;6(1)e13. doi:10.1017/cts.2021.884
7. Bennett LM, Gadlin H. Collaboration and team science: From theory to practice. Conference Paper. J Investig Med. 2012;60(5):768-775. doi:10.2310/JIM.0b013e318250871d
8. McCormack WT, Levites Strekalova YA. CTS teams: a new model for translational team training and team science intervention. J Clin Transl Sci. 2021;5(1):e183. doi:10.1017/cts.2021.854
9. Calhoun WJ, Wooten K, Bhavnani S, Anderson KE, Freeman J, Brasier AR. The CTSA as an exemplar framework for developing multidisciplinary translational teams. Clin Transl Sci. Feb 2013;6(1):60-71. doi:10.1111/cts.12004
10. Osarogiagbon RU, Rodriguez HP, Hicks D, et al. Deploying Team Science Principles to Optimize Interdisciplinary Lung Cancer Care Delivery: Avoiding the Long and Winding Road to Optimal Care. J Oncol Pract. Nov 2016;12(11):983-991. doi:10.1200/jop.2016.013813
11. Benfield MPJ, Utley DR. Describing team development in science and engineering organizations. In: 26th Annual National Conference of the American Society for Engineering Management 2005 - Organizational Transformation: Opportunities and Challenges, ASEM 2005. 2005:350-354.
12. Tomek S. Developing a multicultural, cross-generational, and multidisciplinary team: An introduction for civil engineers. Article. Leadersh Manage Eng. 2011;11(2):191-196. doi:10.1061/(ASCE)LM.1943-5630.0000119
13. Morgan SE, Mosser A, Ahn S, et al. Developing and evaluating a team development intervention to support interdisciplinary teams. Article. J Clin Transl Sci. 2021;5(1)e166. doi:10.1017/cts.2021.831
14. Zucker D. Developing your career in an age of team science. Conference Paper. J Investig Med. 2012;60(5):779-784. doi:10.2310/JIM.0b013e3182508317
15. Shuffler ML, Diazgranados D, Maynard MT, Salas E. DEVELOPING, SUSTAINING, AND MAXIMIZING TEAM EFFECTIVENESS: AN INTEGRATIVE, DYNAMIC PERSPECTIVE OF TEAM DEVELOPMENT INTERVENTIONS. Acad Manag Ann. Jun 2018;12(2):688-724. doi:10.5465/annals.2016.0045
16. Hager K, St Hill C, Prunuske J, Swanoski M, Anderson G, Lutfiyya MN. Development of an interprofessional and interdisciplinary collaborative research practice for clinical faculty. J Interprof Care. 2016;30(2):265-7. doi:10.3109/13561820.2015.1092951

17. Delise LA, Gorman CA, Brooks AM, Rentsch JR, Steele-Johnson D. The effects of team training on team outcomes: A meta-analysis. Article. Perform Improv Q. 2010;22(4):53-80. doi:10.1002/piq.20068
18. Treise D, Baralt C, Birnbrauer K, Krieger J, Neil J. Establishing the need for health communication research: best practices model for building transdisciplinary collaborations. Article. J Appl Commun Res. 2016;44(2):194-198. doi:10.1080/00909882.2016.1155729
19. Shuffler ML, Salas E, Rosen MA. The Evolution and Maturation of Teams in Organizations: Convergent Trends in the New Dynamic Science of Teams. Article. Front Psychol. 2020;112128. doi:10.3389/fpsyg.2020.02128
20. Dogba MJ, Menear M, Stacey D, Brière N, Légaré F. The evolution of an interprofessional shared decision-making research program: Reflective case study of an emerging Paradigm. Article. Int J Integr Care. 2016;16(July-September)4. doi:10.5334/ijic.2212
21. Wooten KC, Calhoun WJ, Bhavnani S, Rose RM, Ameredes B, Brasier AR. Evolution of Multidisciplinary Translational Teams (MTTs): Insights for Accelerating Translational Innovations. Article. Clin Transl Sci. 2015;8(5):542-552. doi:10.1111/cts.12266
22. Salazar MR, Lant TK. Facilitating innovation in interdisciplinary teams: The role of leaders and integrative communication. Article. Inform Sci. 2018;21:157-178. doi:10.28945/4011
23. Ho E, Jeon M, Lee M, et al. Fostering interdisciplinary collaboration: A longitudinal social network analysis of the NIH mHealth Training Institutes. J Clin Transl Sci. 2021;5(1):e191. doi:10.1017/cts.2021.859
24. Ajayi TB, Childs E, Di Frances Remein C, et al. Fostering Tobacco Regulatory Team Science through a multisite, virtual fellowship program for early-career researchers. Article. J Clin Transl Sci. 2022;6(1)e14. doi:10.1017/cts.2021.887
25. Hall KL, Vogel AL, Stipelman BA, Stokols D, Morgan G, Gehlert S. A four-phase model of transdisciplinary team-based research: Goals, team processes, and strategies. Article. Transl Behav Med. 2012;2(4):415-430. doi:10.1007/s13142-012-0167-y
26. Begg MD, Bennett LM, Cicutto L, et al. Graduate Education for the Future: New Models and Methods for the Clinical and Translational Workforce. Article. Clin Transl Sci. 2015;8(6):787-792. doi:10.1111/cts.12359
27. Kirk-Lawlor N, Allred S. Group Development and Integration in a Cross-Disciplinary and Intercultural Research Team. Article. Environ Manage. 2017;59(4):665-683. doi:10.1007/s00267-016-0809-9
28. Schnapp LM, Rotschy L, Hall TE, Crowley S, O'Rourke M. How to talk to strangers: Facilitating knowledge sharing within translational health teams with the Toolbox dialogue method. Article. Transl Behav Med. 2012;2(4):469-479. doi:10.1007/s13142-012-0171-2
29. Dozier AM, Martina CA, O'Dell NL, et al. Identifying Emerging Research Collaborations and Networks: Method Development. Article. Eval Health Prof. 2014;37(1):19-32. doi:10.1177/0163278713501693
30. Fiscella K, Mauksch L, Bodenheimer T, Salas E. Improving Care Teams' Functioning: Recommendations from Team Science. Article. Jt Comm J Qual Patient Saf. 2017;43(7):361-368. doi:10.1016/j.jcjq.2017.03.009
31. Vogel AL, Feng A, Oh A, et al. Influence of a National Cancer Institute transdisciplinary research and training initiative on trainees' transdisciplinary research competencies and scholarly productivity. Transl Behav Med. Dec 2012;2(4):459-68. doi:10.1007/s13142-012-0173-0
32. Knobloch MJ, McKinley L, Keating J, Safdar N. Integrating antibiotic stewardship and infection prevention and control programs using a team science approach. Am J Infect Control. Aug 2021;49(8):1072-1074. doi:10.1016/j.ajic.2021.01.020
33. Begerowski SR, Traylor AM, Shuffler ML, Salas E. An integrative review and practical guide to team development interventions for translational science teams: One size does not fit all. Review. J Clin Transl Sci. 2021;5(1)e198. doi:10.1017/cts.2021.832

34. Rolland B, Cross JE, Hohl SD, Johnson LJ, Wooten K, Brasier AR. Introduction to the themed issue on the design, development, evaluation, and dissemination of team science interventions in clinical and translational research. Review. J Clin Transl Sci. 2021;5(1)e202. doi:10.1017/cts.2021.870
35. Schmitz KH, Bavendam T, Brady SS, et al. Is the juice worth the squeeze? Transdisciplinary team science in bladder health. Neurourol Urodyn. Jun 2020;39(5):1601-1611. doi:10.1002/nau.24357
36. Collaco JM, St Geme JW, 3rd, Abman SH, Furth SL. It Takes a Team to Make Team Science a Success: Career Development within Multicenter Networks. J Pediatr. Jan 2023;252:3-6.e1. doi:10.1016/j.jpeds.2022.08.040
37. Magrane D, Khan O, Pigeon Y, Leadley J, Grigsby RK. Learning about teams by participating in teams. Article. Acad Med. 2010;85(8):1303-1311. doi:10.1097/ACM.0b013e3181e5c07a
38. Gosselin DC, Thompson K, Pennington D, Vincent S. Learning to be an interdisciplinary researcher: incorporating training about dispositional and epistemological differences into graduate student environmental science teams. Article. J Environ Stud Sci. 2020;10(3):310-326. doi:10.1007/s13412-020-00605-w
39. Drotar D. Lessons learned from a career in clinical research: Implications for mentoring and career development. Article. Prof Psychol Res Pract. 2013;44(6):384-390. doi:10.1037/a0035228
40. Fink-Samnick E. Leveraging Interprofessional Team-Based Care Toward Case Management Excellence: Part 2, Team Development, Interprofessional Team Activation, and Sustainability. Article. Professional Case Management. Jan-Feb 2020;25(1):5-18. doi:10.1097/NCM.0000000000000393
41. Largent DL. Measuring and understanding team development by capturing self-assessed enthusiasm and skill levels. Article. ACM J Trans Comput Educ. 2016;16(2)6. doi:10.1145/2791394
42. Weinberger M, Pusek SN, Esserman DA, et al. A model for developing, evaluating, and disseminating best practices in education and training. Article. Clin Transl Sci. 2014;7(5):402-405. doi:10.1111/cts.12189
43. Hall KL, Feng AX, Moser RP, Stokols D, Taylor BK. Moving the science of team science forward: collaboration and creativity. Am J Prev Med. Aug 2008;35(2 Suppl):S243-9. doi:10.1016/j.amepre.2008.05.007
44. Ameredes BT, Hellmich MR, Cestone CM, et al. The Multidisciplinary Translational Team (MTT) Model for Training and Development of Translational Research Investigators. Article. Clin Transl Sci. 2015;8(5):533-541. doi:10.1111/cts.12281
45. Guise JM, Winter S, Fiore SM, Regensteiner JG, Nagel J. Organizational and training factors that promote team science: A qualitative analysis and application of theory to the National Institutes of Health's BIRCWH career development program. J Clin Transl Sci. Apr 2017;1(2):101-107. doi:10.1017/cts.2016.17
46. Zajac S, Woods A, Tannenbaum S, Salas E, Holladay CL. Overcoming Challenges to Teamwork in Healthcare: A Team Effectiveness Framework and Evidence-Based Guidance. Article. Front Commun. 2021;6606445. doi:10.3389/fcomm.2021.606445
47. Milojevic S. Principles of scientific research team formation and evolution. Article. Proc Natl Acad Sci U S A. 2014;111(11):3984-3989. doi:10.1073/pnas.1309723111
48. Bruzzese JM, Usseglio J, Goldberg J, Begg MD, Larson EL. Professional development outcomes associated with interdisciplinary research: An integrative review. Nurs Outlook. Jul-Aug 2020;68(4):449-458. doi:10.1016/j.outlook.2020.03.006
49. Hohl SD, Neuhouser ML, Thompson B. Re-orienting transdisciplinary research and community-based participatory research for health equity. Article. J Clin Transl Sci. 2022;6(1)e22. doi:10.1017/cts.2022.15
50. Vaughan R, Romanick M, Brassil D, et al. The Rockefeller Team Science Leadership training program: Curriculum, standardized assessment of competencies, and impact of returning assessments. Article. J Clin Transl Sci. 2021;5(1)e165. doi:10.1017/cts.2021.838
51. Yu S, Bedru HD, Lee I, Xia F. Science of Scientific Team Science: A survey. Review. Comput Sci Rev. 2019;31:72-83. doi:10.1016/j.cosrev.2018.12.001

52. Salas E, Reyes DL, McDaniel SH. The science of teamwork: Progress, reflections, and the road ahead. Article. American Psychologist. 2018;73(4):93-600. doi:10.1037/amp0000334
53. Salas E, Tannenbaum SI, Kraiger K, Smith-Jentsch KA. The Science of Training and Development in Organizations: What Matters in Practice. Psychol Sci Public Interest. Jun 2012;13(2):74-101. doi:10.1177/1529100612436661
54. Ryan D, Emond M, Lamontagne ME. Social network analysis as a metric for the development of an interdisciplinary, inter-organizational research team. Article. J Interprof Care. 2014;28(1):28-33. doi:10.3109/13561820.2013.823385
55. Levites Strekalova YA, Qin Y, McCormack WT. Strategic Team Science: Scaffolded training for research self-efficacy, interdisciplinarity, diversity, equity, and inclusive excellence in biomedical research. J Clin Transl Sci. 2021;5(1):e195. doi:10.1017/cts.2021.810
56. Meyers FJ, Begg MD, Fleming M, Merchant C. Strengthening the Career Development of Clinical Translational Scientist Trainees: A Consensus Statement of the Clinical Translational Science Award (CTSA) Research Education and Career Development Committees. Article. Clin Transl Sci. 2012;5(2):132-137. doi:10.1111/j.1752-8062.2011.00392.x
57. Porter SG, Smith TM. Teaching Team Science Through Hackathons. Letter. Omics : a journal of integrative biology. 2022;26(9):521-522. doi:10.1089/omi.2022.0102
58. Lacerenza CN, Marlow SL, Tannenbaum SI, Salas E. Team development interventions: Evidence-based approaches for improving teamwork. Am Psychol. May-Jun 2018;73(4):517-531. doi:10.1037/amp0000295
59. Liu M, Jaiswal A, Bu Y, et al. Team formation and team impact: The balance between team freshness and repeat collaboration. Article. J Inf. 2022;16(4)101337. doi:10.1016/j.joi.2022.101337
60. Bergey P, King M. Team machine: A decision support system for team formation. Article. Decis Sci J Innovative Educ. 2014;12(2):109-130. doi:10.1111/dsji.12027
61. Campbell-Voytal K, Daly JM, Nagykaldi ZJ, et al. Team Science Approach to Developing Consensus on Research Good Practices for Practice-Based Research Networks: A Case Study. Article. Clin Transl Sci. 2015;8(6):632-637. doi:10.1111/cts.12363
62. Santillan MK, Becker RC, Calhoun DA, et al. Team Science: American Heart Association's Hypertension Strategically Focused Research Network Experience. Hypertension. Jun 2021;77(6):1857-1866. doi:10.1161/hypertensionaha.120.16296
63. Johnston JH, Phillips HL, Milham LM, et al. A team training field research study: Extending a theory of team development. Article. Front Psychol. 2019;10(JUN)1480. doi:10.3389/fpsyg.2019.01480
64. Antonucci TC. Teams Do It Better! Article. Res Hum Dev. 2015;12(3-4):342-349. doi:10.1080/15427609.2015.1068035
65. Donesky D, Anderson WG, Joseph RD, Sumser B, Reid TT. TeamTalk: Interprofessional Team Development and Communication Skills Training. Article. J Palliat Med. 2020;23(1):40-47. doi:10.1089/jpm.2019.0046
66. Libby AM, Cornfield DN, Abman SH. There Is No "I" in Team: New Challenges for Career Development in the Era of Team Science. Editorial. J Pediatr. 2016;177:4-5. doi:10.1016/j.jpeds.2016.06.082
67. Shuffler ML, DiazGranados D, Salas E. There's a science for that: Team development interventions in organizations. Review. Current Directions in Psychological Science. 2011;20(6):365-372. doi:10.1177/0963721411422054
68. Love HB, Fosdick BK, Cross JE, et al. Towards understanding the characteristics of successful and unsuccessful collaborations: a case-based team science study. Article. Hum Soc Sci Comm. 2022;9(1)371. doi:10.1057/s41599-022-01388-x
69. Kelly TH, Mattacola CG. Training and career development in clinical and translational science: An opportunity for rehabilitation scientists. Article. J Sport Rehab. 2010;19(4):369-379. doi:10.1123/jsr.19.4.369

70. Knowlton AA, Rainwater JA, Chiamvimonvat N, et al. Training the translational research teams of the future: UC Davis-HHMI integrating medicine into basic science program. Article. Clin Transl Sci. 2013;6(5):339-346. doi:10.1111/cts.12068
71. Clark MA, Rogers ML, Boergers J, et al. A transdisciplinary approach to protocol development for tobacco control research: A case study. Article. Transl Behav Med. 2012;2(4):431-440. doi:10.1007/s13142-012-0164-1
72. Klenk NL, Meehan K. Transdisciplinary sustainability research beyond engagement models: Toward adventures in relevance. Article. Environmental Science and Policy. 2017;78:27-35. doi:10.1016/j.envsci.2017.09.006
73. Czajkowski SM, Lynch MR, Hall KL, et al. Transdisciplinary translational behavioral (TDTB) research: opportunities, barriers, and innovations. Transl Behav Med. Mar 2016;6(1):32-43. doi:10.1007/s13142-015-0367-3
74. Wooten KC, Dann SM, Finnerty CC, Kotarba JA. Translational Science Project Team Managers: Qualitative Insights and Implications from Current and Previous Postdoctoral Experiences. Postdoc J. Jul 2014;2(7):37-49.
75. Yousefi Nooraie R, White RJ, Steele S, Augustine EF, Ossip DJ, Zand MS. Un-Meetings as tools for translational idea generation: A semantic content analysis of an Opioid Crisis Un-Meeting. J Clin Transl Sci. 2022;6(1):e124. doi:10.1017/cts.2022.490
76. Lester J, Kezar AJ. Understanding the Formation, Functions, and Challenges of Grassroots Leadership Teams. Article. Innovative High Educ. 2012;37(2):105-124. doi:10.1007/s10755-011-9191-y
77. Adler NE, Stewart J. Using team science to address health disparities: MacArthur network as case example. Blackwell Publishing Inc.; 2010. https://www.scopus.com/inward/record.uri?eid=2-s2.0-77249166619&doi=10.1111%2fj.1749-6632.2009.05335.x&partnerID=40&md5=ef8ee22ccb8c2fa88e169d59cb34b149
78. Haynes NJ, Vandenberg RJ, DeJoy DM, et al. The workplace health group: A case study of 20 years of multidisciplinary research. Am Psychol. Apr 2019;74(3):380-393. doi:10.1037/amp0000445
