# Supplementary material for: Temporal development of high-performance translational teams
Source: J Clin Transl Sci. 2023 May 15;7(1):e117. doi: 10.1017/cts.2023.545 (PMC10225261; doi:10.1017/cts.2023.545)
Supplement: Supplementary file 1 [file ctssup.zip › S2059866123005459sup002.pdf]

**Scoping review protocol:**

(Following JBI Manual for Evidence Synthesis: Peters MDJ, Godfrey C, McInerney P, Munn Z, Tricco AC, Khalil, H. Chapter 11: Scoping Reviews (2020 version). In: Aromataris E, Munn Z (Editors). JBI Manual for Evidence Synthesis, JBI, 2020. Available from https://synthesismanual.jbi.global. https://doi.org/10.46658/JBIMES-20-12

**Title:** Developmental phases of translational teams

**Introduction:** Development phases that translational teams master from preclinical to clinical development are not well understood. A review to identify existing and appropriate phases is needed.

**Definitions**

- Translational Team (TT) -an interdisciplinary team that seeks to improve human health by developing a device, intervention, drug, diagnostic for sustainable adoption and use .
- Competencies: knowledge, skills and attitudes demonstrated by a team.
- Team-emergent competencies: KSAs arising from team interactions.
- Performance-advancement across the translational research spectrum.
- Affect-a domain describing that the bonds between TT members are grounded in a concern, empathy and shared regard for others.
- Communication- a KSA where team members effectively exchanges information and integrates team member expertise to solve research problems.
- Management - actions that effectively organize and sustain components of multicomponent investigation.
- Collaborative problem solving- refers to situation where cognitive and social skills of the TT are used to integrate research findings and discipline-grounded interpretations into a cohesive model.
- Leadership- the process providing or supporting the cognitive, resource and affective needs for a TT.
- Development- discrete and identifiable states of a TT

**Details of preliminary work/searches-**

1. Kara L. Hall, PhD, Amanda L Vogel, PhD, MHS, Brooke A Stipelman, PhD, Daniel Stokols, PhD, Glen Morgan, PhD, Sarah Gehlert, PhD, A four-phase model of transdisciplinary team-based research: goals, team processes, and strategies, Translational Behavioral Medicine, Volume 2, Issue 4, December 2012, Pages 415–430, https://doi.org/10.1007/s13142-012-0167-y.

This paper is an essay drawing from the authors experiences that include perspectives in team science, psychology and organizational theory. 67 primary and review papers are cited. Search strategy is not described.

**Overall review objective**- Identify what specific behaviors or skills in these competency domains most directly impact TT performance?

**Search strategy used for this review:**

Databases Searches

Scopus

64 Results

((TITLE-ABS-KEY(Transdisciplin* OR trans-disciplin* OR cross-disciplin* OR team-science OR team-based-research)) AND (TITLE(group-development OR formation OR maturation OR

sequential-development OR punctuated-equilibrium)) AND PUBYEAR > 2009) OR (TITLE-ABS-KEY(((cross-disciplin* OR transdisciplin* OR interdisciplin* OR inter-disciplin* OR translational) AND (team*)) OR team-science) AND TITLE-ABS-KEY(development* OR formation OR maturation)) OR (((TITLE(Transdisciplin* OR trans-disciplin* OR cross-disciplin* OR ((team*) W/3 (science)) OR ((team-based) W/2 (research))))) AND ((TITLE-ABS-KEY(((group OR sequential) W/3 (development)) OR (punctuated W/2 equilibrium*) OR (development W/3 model*)))) AND PUBYEAR > 2009) AND ( LIMIT-TO ( EXACTSRCTITLE,"Journal Of Clinical And Translational Science" ) OR LIMIT-TO ( EXACTSRCTITLE,"Clinical And Translational Science" ) OR LIMIT-TO ( EXACTSRCTITLE,"Translational Behavioral Medicine" ) ) AND ( LIMIT-TO ( LANGUAGE,"English" ) ) AND ( LIMIT-TO ( DOCTYPE,"ar" ) OR LIMIT-TO ( DOCTYPE,"re" ) )

Web of Science (Medline, Core Collection, Current Contents)

Limited to Articles/Reviews only

184 results

(((TS=(((cross-disciplin* OR transdisciplin* OR interdisciplin* OR inter-disciplin* OR translational) AND (team*)) OR team-science) AND TS=(development* OR formation OR maturation)) AND SO=(JOURNAL OF CLINICAL AND TRANSLATIONAL SCIENCE or CTS CLINICAL AND TRANSLATIONAL SCIENCE or ANNALS OF TRANSLATIONAL MEDICINE or TRANSLATIONAL BEHAVIORAL MEDICINE)) OR ((TI=(Transdisciplin* OR trans-disciplin* OR cross-disciplin* OR ((team*) NEAR/3 (science)) OR ((team-based) NEAR/2 (research))))) AND ((TS=(((group OR sequential) NEAR/3 (development)) OR (punctuated NEAR/2 equilibrium*) OR (development NEAR/3 model*)))) OR ((TS=(Transdisciplin* OR trans-disciplin* OR cross-disciplin* OR team-science OR team-based-research)) AND (TI=(group-development OR formation OR maturation OR sequential-development OR punctuated-equilibrium)))) AND LA=(english) AND PY=(2010-2023)

Backward citation chaining

Begerowski SR, Traylor AM, Shuffler ML, Salas E. An integrative review and practical guide to team development interventions for translational science teams: One size does not fit all. J Clin Transl Sci. 2021;5(1):e198. Published 2021 Aug 9. doi:10.1017/cts.2021.832

**Eligibility criteria for inclusion into the analysis:**

1. Study type: Empiric-observational; Empiric-survey, Meta-analysis, or Expert opinion/panel
2. Analysis or description of developmental phases
3. Team-type relevance: Knowledge-generating, product development, innovation, translational team

**Selection and classification of articles.** Article abstracts were screened by the primary author (a list of all 78 considered is provided in **Supplemental File #2**).

**Conflict resolution-** negotiation among authors

**How data will be presented** – Papers are cited and summarized in text.
